# Diversifying audiences and producers of public involvement in scientific research: the AudioLab

**DOI:** 10.1186/s40900-018-0122-2

**Published:** 2018-11-12

**Authors:** Bella Starling, Jemma Tanswell

**Affiliations:** 1 Public Programmes, Research & Innovation, Manchester University NHS Foundation Trust, University of Manchester, Manchester Academic Health Science Centre, Manchester, M13 9WU UK; 2 Reform Radio CIC, 2 Atherton St, Manchester, M3 3BE UK

**Keywords:** Public, Patient, Young adult, Involvement, Engagement, Diversity, Inclusion, Science, Communication, Creative

## Abstract

**Plain English summary:**

A partnership between a public engagement specialist, and a director of a digital radio station working towards social change, The AudioLab is a creative and innovative way to support diverse young people to connect with and get actively involved in health research. We worked with 25 creative young adults at relative socioeconomic, educational and/or health disadvantage in 2016 and 2017. Facilitated by the project leads, the emerging young talents connected with science in a series of culturally- and personally-relevant and creative sessions, culminating in their production of digital content and a one-hour live radio show, which reached at least 2324 people online. As a result of taking part in The AudioLab, the young adults felt more confident about themselves and about science; they could see a role for their creative talents in science communication, and felt able to become actively involved in health research. After the project, the ongoing partnership between the project leads and the young adults helped 12 of the participants to progress to employment, training and voluntary roles within health, research, creative and communications environments. We believe that the AudioLab presents a way of working that takes a step towards greater diversity and inclusion among both the audiences and producers of public involvement in health research. It has the potential to be reproduced as a method in other locations.

**Abstract:**

**Background**

The AudioLab is an innovative way to support greater inclusion in public engagement and involvement in health research. A partnership between a public engagement with research specialist, and a director of a digital radio station working towards social change, The AudioLab piloted an approach working with young people at relative socioeconomic, educational and/or health disadvantage to engage and involve them meaningfully, and sustainably, with health science and research.

**Methods**

Twenty-five young adults from diverse backgrounds took part in AudioLab pilots in 2016 and 2017. Facilitated by the project leads, they engaged with science in a series of culturally-relevant and creative sessions, culminating in their production of digital content and a one-hour live radio show, the latter reaching at least 2324 people online.

**Results**

Young adults’ agency within health research and science engagement was increased, with 12 of the participants progressing to roles within health, research, creative and communications environments.

**Conclusions**

Through partnership working between the project leads, co-production of creative outputs by the young people with scientists and science communicators, and a ‘reimagining’ of the positive assets that diverse young adults bring to engagement with health research, the AudioLab presents a way of working that takes a step towards greater diversity and inclusion among both the audiences and producers of public involvement in health research. Further, the AudioLab has the potential to be reproduced as a method in other locations.

**Electronic supplementary material:**

The online version of this article (10.1186/s40900-018-0122-2) contains supplementary material, which is available to authorized users.

## Background

### Diversity and inclusion in public involvement in scientific research

Diversity and inclusion in public involvement in research has become a pressing concern in the last few years. ‘Going the Extra Mile’ [1], the National Institute of Health Research’s strategic review of public involvement in the UK, states that “a diverse and inclusive public involvement community is essential if research is relevant to population needs and provides better health outcomes for all.” Likewise, the Wellcome Trust’s priority area on Diversity and Inclusion [2] includes the need for “engaging a wider range of people with science and health”.

How we become more socially inclusive in science and research engagement is an evolving and shifting field of practice and theory. It is not a new concern; however, relatively little has been published in this area. A review for the Wellcome Trust [3] identified 14 studies focusing on patient and public involvement and diversity and inclusion. The same report draws attention to the fact that reporting and data capture related to diversity and inclusion in public involvement in research is challenging and lacking. A special issue of Journal of Science Communication has focused on socially inclusive science communication [4] and some articles in this journal have also focused on the agenda [5].

### Why the need for greater diversity and inclusion in public involvement in scientific research?

Diversity is important for innovation in science. Increased productivity, competitiveness and creativity through inclusion are cited as ‘business’ advantages for science [3]: arguably more inclusive public involvement can lead to more inclusive and relevant science. By extension, more inclusive public involvement has the potential to stimulate innovation in its own practices.

Inclusion supports social and health justice and equity. Addressing the relevance and priorities of research to disadvantaged and/or marginalised groups through inclusive engagement, as well as how the outputs of research have the potential to positively impact on their health, enhances the ethics and governance of research [6].

The Universal Declaration of Human Rights (article 27) [7] affirms that access to scientific information is a human right; equitable participation and fair access underpin a rights-based approach to public engagement with science.

Opening up science practices to be more socially inclusive is a component part of a deliberative, participatory democracy contributing to policy decisions (in science, health and other areas) [8]. Sharing, democratising and co-producing knowledge has individual as well as collective political value, often benefiting people through increased knowledge, confidence, skills and agency. As Jean Perrin said in 1937 at the opening of the Palais de la Decouverte in Paris, “will ensure the progressive liberation of all human beings and [...] the possibility to open to everyone the joy of art and thought”.

### Science communication, public engagement and patient involvement

The authors of this paper acknowledge some real and perceived differences in the aims, drivers and activities of ‘science communication’ [9], ‘public engagement’ ([10], National Coordinating Centre for Public Engagement]) and ‘public and patient involvement in research’ ([11], INVOLVE]) as well as acknowledging overlapping areas of ambition and commonality. In this paper, we adopt a broad definition and shorthand of ‘public involvement in scientific research’ to mean all the ways in which people can connect with and work together in scientific research, not least because, from a public point of view arguably, the distinctions are artificial. Where necessary, we draw distinctions between areas of practice. Common to all fields, however, are calls for greater diversity and inclusion in both audiences and producers of public involvement with science.

### Moving beyond a ‘barriers’ approach to public involvement in scientific research

On a large scale, surveys such as the UK Public Attitudes to Science [12], the Wellcome Trust Monitor [13] and the Health Research Authority [14] characterise people engaged (or not) with science. Smaller scale public engagement and involvement initiatives routinely monitor and evaluate the ‘who’, ‘how’ and impact of engagement, even if more granular data about diversity and inclusion are relatively lacking [3]. The UK Public Attitudes to Science identifies ‘concerned’ and ‘disengaged sceptics’ who feel ill-informed about science, do not trust science or scientific regulation and rarely participate in science communication activities. These descriptors include higher proportions of people from minority ethnic backgrounds, socio-economically disadvantaged backgrounds and women. This framing is unhelpful [15] reinforcing negative constructions of people and identities, perhaps feeling marginalised already, who might not wish to participate in science. It does not take into account the social contexts of scientists nor of the people who engage with science.

The Health Research Authority report highlights that only 35% of people from ethnic minorities are confident that they would be treated with dignity and respect if they took part in health research, compared to 50% of white respondents. Similarly, 59% of those in social class AB were very confident in health research as opposed to 39% of those in social class DE; and 56% of people who had completed a degree or higher were very confident in comparison with just 36% of people with no formal qualifications. Arguably the patient involvement sector is moving towards a recognition of the value and diversity of lived experience of health and research, though this is not widespread [16].

Emily Dawson argues compellingly for a re-evaluation of how we ‘imagine’ people engaged in science to move beyond a ‘barriers’ approach to public engagement, in which lack of access or lack of interest are simple defining features [17]. She argues for a more complex view of exclusion of certain publics from engagement with science, which includes acknowledgement of structural inequalities arising from cultural imperialism and powerlessness [18] as well as issues related to intersectionality, for example race/ethnicity and gender. “Social reproduction in science communication constructs a narrow public that reflects the shape, values and practices of dominant groups, at the expense of the marginalised”: why should someone engage with a thing - science - that feels like it just relates to those with power in society?

As Dawson summarises: “the challenge then, for researchers, practitioners, funders and policy makers lies in how to understand and address the complex, multiple and embedded issues involved in social exclusion from science communication” [19]. The challenge is not just to re-imagine people engaged in science, but science communication itself.

### Aims of the AudioLab

The starting premise for our work on The AudioLab project was that not only does diversity improve innovation in science, but also that diversity and inclusion improves public involvement in science. Moreover, that public involvement in science is important for a fair and just society, by developing transferable confidence, skills and agency in science and public involvement in people who might feel excluded by traditional science culture or mainstream society.

The AudioLab worked with young adults seeking employment, with creative aspirations. Sometimes referred to as being at relative educational, health and/or socioeconomic disadvantage or young people out of education, employment or training, the co-authors have chosen not to use this terminology, preferring an approach that does not stigmatise. This group are an underrepresented constituency in science engagement [20]. We set out to engage young adults in a way that was driven by the young adults themselves, relevant to their everyday lives, to their cultural environment, their future aspirations and building on their positive social identities and assets.

Our aims were to:

- Engage, in depth, young adults seeking employment with scientific research;
- Enable young adults seeking employment with scientists and/or public involvement practitioners to co-produce engaging audio/digital content about scientific research;
- Engage a wider audio audience with science in a relevant way;
- Experiment with an engagement approach building on concepts of science capital and cultural democracy;
- Support science researchers and public engagement practitioners to engage with audiences who might not see science as ‘for them’.

### Conceptual and practical underpinnings to the AudioLab

We adopted an action learning approach to reimagine the methods, spaces and voices needed to overturn the dominant values and practices of public engagement with science, so as to become more inclusive. Our approach built on concepts of ‘Science Capital’, cultural democracy and chose to work through an accessible and democratic medium: radio.

Science Capital [21] is a social justice framework which addresses why particular groups remain underrepresented in science. It aims to provide a measure of engagement with science to ascertain how someone might feel science is ‘for them’. It identifies 8 dimensions - scientific literacy; science related attitudes, values and dispositions; knowledge about the transferability of science; consumption of science-related media; participation in learning activities; family science skills, knowledge and qualifications; knowing people in science related roles and jobs; talking to others about science in everyday life - which mould a person’s connection with science, and can be strengthened through interventions. Building a person’s science capital will hopefully have a positive effect on their lives, not just by encouraging a connection with science, but by improving people’s lives and life chances.

Cultural democracy focuses on the importance of everyday creativity, the promotion of cultural diversity, and the right to culture for everyone beyond access to publicly funded art [22]. It places culture made by all as a vital part of community life. In a recent report [22], radio is highlighted as a useful and democratic medium for cultural democracy. It is a flexible and person-centred format, which is accessible both in terms of audience and content production.

Community radio is a non-profit activity, owned, managed and controlled by local communities. In theory therefore, it offers the potential for more broad-based participation in deliberation and debate within the public sphere engaging multiple voices and perspectives and contributing towards progressive social change [23]. “Radio stations are not simply passive transmitters of information or music; they are a catalyst for building community, for improving health and education, for fostering a civil society. The expressive human voice and natural sound engage the imagination through story telling” [24]. ‘More than Yacking Away’ [25], a review of youth learning opportunities in the community radio sector, highlights that community radio provides a unique learning culture for young people, through equal and participatory spaces. Young people can express themselves and be creative, have the freedom and autonomy to produce programmes that help them feel recognised and represented in their communities.

### The AudioLab partnership

The AudioLab stems from a partnership between the co-authors of this paper. Bella Starling is a public involvement practitioner embedded in the health research environment of Greater Manchester. Her career and current work encompasses science communication, public engagement and patient involvement as Director of the Public Programmes team [26] at Manchester University NHS Trust, part of the NIHR Manchester Biomedical Research Centre. Her Wellcome Engagement Fellowship, which funded this work, focuses on public engagement with health research as a catalyst for social change.

Jemma Tanswell is a Director of Reform Radio [27]. Reform Radio is an innovative radio station and community interest company. As well as cutting edge broadcast content, behind the scenes, a range of creative workshops and digital programmes support individuals to develop transferrable skills for future careers. It focuses on working with young people aged 16–30 seeking employment. Many of the young people coming through Reform’s doors experience challenging socioeconomic, health and/or educational backgrounds.

## Methods - what we did

### Recruitment

#### Inclusion criteria

To be eligible to participate in The AudioLab, you have to be:

- Aged between 18 and 30 years old
- Out of employment, education and/or training
- Living within the Greater Manchester area.

### Recruitment process

**Fig. 1:**
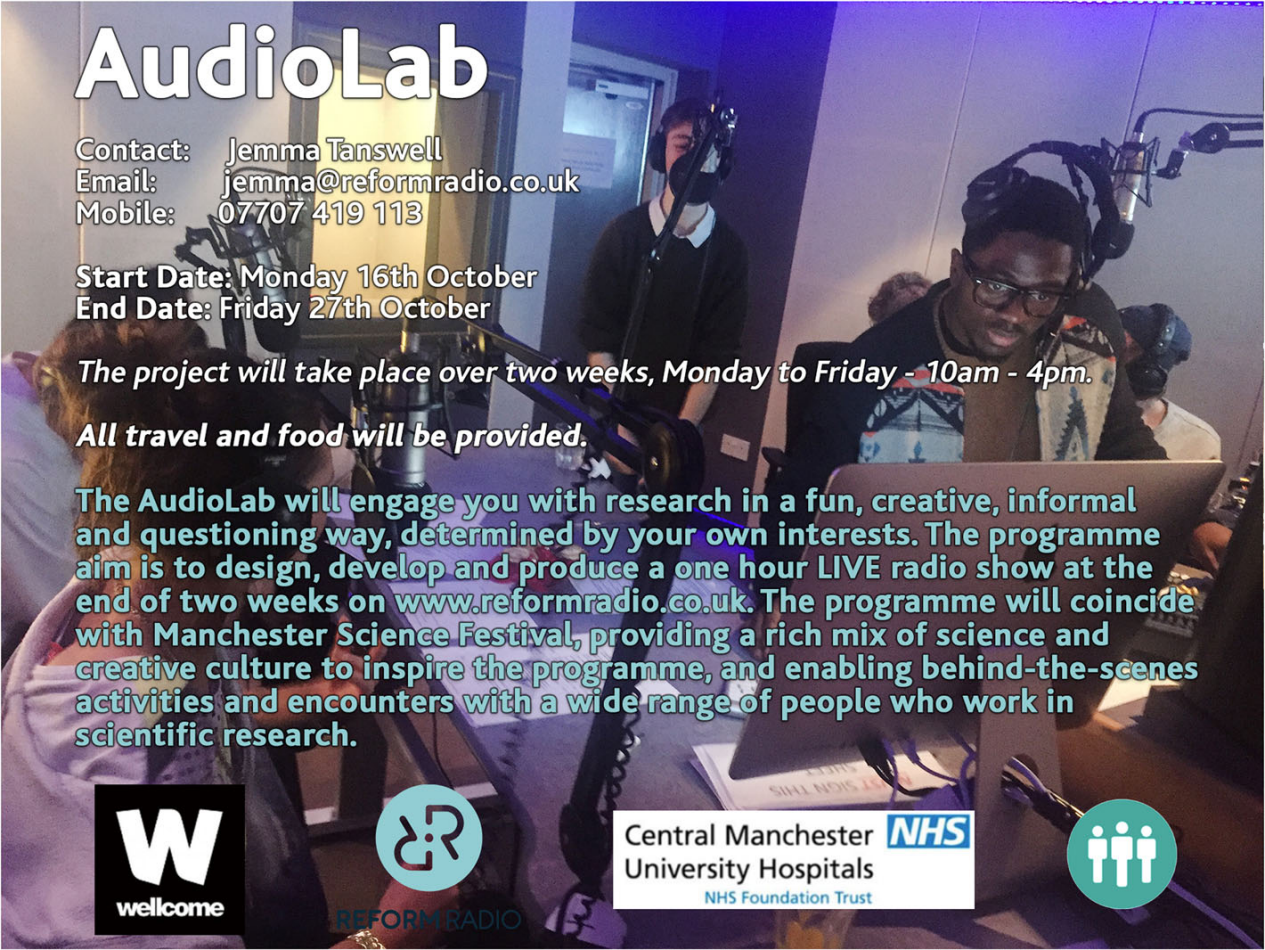
Flyer used to promote and recruit to The AudioLab

Young adults were recruited to The AudioLab through Reform Radio’s existing relationships with a wide range of community and voluntary sector, social change, youth work and employment organisations, such as Job Centres, Pupil Referral Units, local mental health charities (eg. Manchester MIND), the Prince’s Trust, Street League, Manchester Adult Education Service and the National Probation Service. The opportunity to take part in the The AudioLab was advertised through distribution of the project flyer (Fig. 1) to the partner organisations listed above and by referrals of potential participants.

Reform colleagues followed up referrals with personal contact with the potential participant (on occasion, with the participant’s family members or agency staff) to explain the programme in a bit more detail, and offer solutions to mitigate challenges in attending: for example, mapping out the best route to get to the project location, paying public transport costs upfront, paying for small amounts of phone credit in order to remind the participant, on the day, of their plan and desire to take part in The AudioLab, once signed up. Participation in The AudioLab was entirely voluntary. Travel expenses and a £3 lunch allowance were provided to each participant.

- 13 young adults took part in 2016;
- 12 young adults took part in 2017; two left the project half way through.

Participation data routinely recorded by Reform Radio for reporting purposes showed the demographic of the groups Table 1.

**Table 1:** Characteristics of young adults taking part in the AudioLab

	2016	2017
Greater Manchester Boroughs (*)	11	10
Age	13 × 19–30 year olds	10 × 19–30 year olds 2 aged 30+ (**)
Ethnicities	3	8
Genders	7 male, 4 female	6 male, 6 female

Cohorts included members with lived experience of mental and/or physical health conditions, homelessness, carers, a care leaver, an ex-offender, and refugee

(*) Greater Manchester comprises 10 Boroughs. In the 2016 cohort, one participant came from a Borough directly adjacent to Greater Manchester

(**) In 2017, the decision was made to include two participants over the age of 30 because of their interest in their own physical health conditions

### The AudioLab pilots

The AudioLab consisted of two pilot programmes in the autumns of 2016 and 2017. Learning from the first informed the second. Each pilot consisted of a series of group and individual creative sessions, structured around creative skills and scientific topics. The culmination of each pilot was broadcast of content created by the young adults about scientific research; the second pilot culminated in a one-hour live radio show, entirely produced by the young adults, and broadcast in Reform Radio’s community slot, which happens every Friday between 2 and 3 pm.

In 2016, 19 creative sessions were run once a week over an 8-week period. Sessions included: a ‘science day’ which included presentations and hands-on activities with various scientists, creative sessions focused on shortform film-making, and cultural experiences (eg. Visit to the Museum of Science and Industry). Working in small groups, the young adults chose to focus on sleep research. They chose this topic because of its resonance with the importance of sleep in their everyday lives. They produced:

- An idea for a Gaming App;
- An idea for a research project on the effects of sleep deprivation on gaming (not vice versa);
- A shortform film on the effect of sleep deprivation on everyday activities and performance;
- A creative shortform documentary on sleep paralysis;
- A Spoken Word piece.

In 2017, 22 creative sessions were run over 10 days, in the run up to and coinciding with the Manchester Science Festival. Creative sessions included spoken word, debate, panel discussion, music, dance, rap battle, hands-on ‘wet’ science activities. The young adults produced a one-hour live radio show, covering various scientific research topics which they picked relevant to their interests developed during the pilot. The radio show (called “Brainwaves”, title chosen by the young adults) can be listened to online (www.reformradio.co.uk/projects/), the running order is shown in Table 2 and a short film (produced entirely by the young adults, with editing support from the project leads) is available as additional material (Additional file 1).

**Table 2:** Running order for the AudioLab’s ‘Brainwaves’ live radio show

Feature	
Introduction to Brainwaves	
Dear Creativity	
Review of Manchester Science Festival	
Documentary and interviews about psychosis, including how to get involved in research	
Performance of ‘DNA’ song, and interview about the process of writing	
Periodic Table battle quiz, pitching science communicators versus AudioLab participants	
Performance of ‘Neuromusicology’ Track inspired by AudioLab sessions	
Performance of ‘Science is Life’ spoken word piece	
Performance of spoken word summary/evaluation of The AudioLab	
Outro & end.	


**Additional file 1:** Short film summarising The AudioLab, made by the young participants of the 2017 cohort. (MP4 305664 kb)


### Evaluation

Evaluation of The AudioLab was both summative and formative, and our approach focused on qualitative and participatory methods, using semi-structured questions and interviews. Interviews with the young adults were carried out by Jemma Tanswell or Bella Starling, or by the young adults themselves, interviewing each other – this also gave the participants the opportunity to ‘practice’ with audio equipment and recording – or as part of group sessions – in some cases, also serving as discussion points for the group. Audio content was transcribed by the project leads, and common themes emerging from the evaluation content were identified by Bella Starling and Jemma Tanswell (with methodology support from Suzanne Parsons cf. Acknowledgements section).

#### Knowledge, attitudes and understanding of science and/or health research amongst participants

Young adults’ experiences and perceptions of science and/or health research were captured at the start, most days during the programme, and the week following The AudioLab pilots. Questions included:

- What do you think about when you think about science?
- What do you feel when you think about science?
- What have you learned?
- Where has science featured today?

#### Knowledge, attitudes and understanding of science communication amongst participants

The views of the young adults taking part in The AudioLab about science communication, public engagement and involvement in scientific research were assessed at the pilot programme mid-point (these were added for the 2017 pilot, as a result of reflection from the 2016 pilot). Questions included:

- What do you think about science communication?
- What skills might be useful to become a science communicator?

#### Format, style and content of the AudioLab programme

The views of the young adults about what worked and what didn’t work about the format, style and content of The AudioLab programmes were captured most days during the pilots and in the week following the pilots. Examples of questions include:

- What did you like about today’s session?
- What would you put in the bin?

#### Participant skills and confidence

The skills, confidence and learning of the participants were assessed, both in relation to science and/or health research and in relation to personal development, at the start, mid-point and after the pilots. Questions included:

- What would you like to learn from this project?
- What do you want to improve?
- What do you want to achieve?
- What was your challenging moment?
- What’s your promise to yourself?

Participants also filled in a grid on a flipchart, capturing (at the start of the pilot) their current skills, which ones they wanted to focus on developing, and (at the end) whether they felt they had achieved them.

#### Summary evaluation

Quantitatively, we assessed the numbers of:

- Young adults taking part in the pilots,
- Engagement and research professionals engaged in the project,
- Numbers of people reached through online and audio content.

In the 2017 pilot, one of the young adults summarised the project from the perspective of a participant, using spoken word (available as part of the “Brainwaves” programme). This was an opportunistic element of the evaluation, based on the skills and outlook of one of the participants and embedded within the pilot itself. Creating this ‘professional’ role within the project ensured their engagement, and provided a unique summary evaluation aspect of the pilot.

#### Progression routes undertaken by the AudioLab participants

Following the pilots, the participating young adults remained under the pastoral care of Reform Radio, with regular meetings, including at 3 and 6 months with one or both AudioLab project leads. These meetings encouraged, assessed and documented progression routes for the young adults, in terms of their creative and employment skills and prospects, including within science communication and public involvement in research.

#### Expectations and experiences of research and engagement professionals

Audio interviews with research and engagement professionals involved in The AudioLab were carried out by the project leads or the young adults, before and after their contributions to the pilots. Questions included:

- What were your expectations of today?
- What went well?
- What didn’t go so well?

#### Project team reflections on pilot format, participant needs and programme direction

At the end of each session day during the pilots, the project leads conducted daily debriefs. This included reflections on what went well, what didn’t go well, key moments, and identified any specific support needed for individual participants. Between the pilots, the project leads met regularly to discuss the progression routes (see below) of participants, reflect on the evaluation findings, identify and plan dissemination opportunities (eg. Conference attendance), future formats and directions for The AudioLab as a programme. These were captured through reflection notes, actions and changes for the pilots and the programme.

## Findings and discussion - what we learned

We base our discussion around the main areas of focus of our evaluation.

### Topics and formats for creative sessions

The AudioLab’s design and delivery incubated and nurtured 25 young adults, from all Boroughs in Greater Manchester and with diverse backgrounds and ethnicities to follow a path of engagement with science. We placed an emphasis on the young adult participants directing topics and formats for engagement. ‘Tasters’ of various scientific areas were provided by the project team initially to guide participant choice, however, the final choice for what the groups focused on in producing their creative content, was up to them. In the 2016 pilot, the whole group chose sleep research because of the relevance of the area to the interests and circumstances of the group. In the 2017 pilot, mental health research, genetics, the periodic table, science and religion, meteorology and space exploration all made an appearance. The diversity of subject areas presented a challenge for the project team in terms of providing creative sessions related to chosen topic areas for the group; however, the experience and connections of the project team enabled this (see below section on Personnel and Partnerships). Enabling this choice and control was felt to be important by the project leads, both to demonstrate to the group that they were being listened to and valued and to capitalise on expressed interests.

A major difference between the 2016 and 2017 pilots was the decrease in timescale of creative sessions (22 over 2 weeks in 2017; 19 over 8 weeks in 2016). We changed the creative sessions format based on the evaluation findings (from both the young adults and project leads) of the 2016 pilot showing a need to keep the momentum of engagement going. We also timed the 2017 pilot to take place during the Manchester Science Festival, to enable greater access to science-related activities.

Evaluation of the creative sessions showed that initially creating a ‘safe space’ for the group was important. This took the form of a number of sessions with just the project leads and focused on participatory and creative exercises, rather than science. Some of the sessions involved external visits and events (eg. A visit to a science exhibition, the ‘Science Day’ in 2016, spoken word events at the Manchester Science Festival in 2017). Evaluation of these showed that traditional formats (exhibitions, scientist talks) were less successful in engaging the young adults than formats in which something other than ‘the wonder of science’ was shared eg. Religion and/or ethnicity of the presenter, creative format (eg. Spoken word events), or formats that were highly participatory, interactive and/or immersive (eg. Virtual reality, games) were particularly valued.

The medium of audio and radio proved itself to be an accessible and democratic format for the young adults to express their creativity about science. The audio equipment was portable and easy to use, and the variety of roles in front of and behind the microphone meant that each participant found something to fit to their talent.

### Increasing ‘science capital’ and personal agency in scientific research

#### Knowledge, attitudes and understanding of science and health research

At the beginning of the project, the young adults typically displayed low ‘Science Capital’. Generally, they were quite intrigued and interested by science, but didn’t see it as ‘for them’. Their perceptions were that it was complex and hard work, inaccessible and several had been ‘turned off’ science at school.

*“I’m curious…. intrigued…. It’s important”*

*“I feel intrigued and, like, curious, ‘cos there’s so many unanswered questions about, like, the earth and the big bang, how humans started, and the planets, and yeah, really intrigued, curious and want to know some answers”*

*“It’s dull…. Makes me nervous… It’s inaccessible….”*

*It’s locked behind journals or in research papers and it’s quite difficult to access”*

*“I think especially when it’s taught to you in schools it can be quite dull”*

*“I think you fall out of it once you finish school, but I think it’s, if anything, probably you have more interest in it after that age”*

[Selection of young adult responses at beginning of pilots]

At the end of the project, the young people were much more ‘turned on’ to science. Participants found science, “*interesting*”, “*worthwhile*”.

*“I’m more interested than I thought”*

*“It’s changed my opinion [about science]”*

“*Science is the past, present and future, it’s not just a topic”*

*“We can be scientists within ourselves, you don’t have to be Einstein to do that”*

[Selection of young adult responses at end of pilots].

As well as learning more about individual topics in science and health research throughout the pilots, the young adults also appreciated elements of the scientific process, the production of evidence and ways to explore the validity of scientific ‘facts’.

*“I’ve learned not to base my perception on just what I think, there’s other sources, your judgement can change”*

*“I’ve learned more about the manipulations, or not really manipulations, but the way in which advertisements [use science to] pull on your heartstrings and your feelings”*

[Selection of young adult responses during the pilots].

#### Participant skills, confidence and agency

During the pilots, young adults developed their own skills and confidence through the creative sessions.

*“I feel more confident in myself […] and science is less scary”*

*“I liked seeing everyone’s confidence come up a little bit”*

[Young adult responses]

Even the less successful creative session formats were useful. On reflection and through discussion with the project leads, the young people understood where their talents and skills could add value to science - by helping science to communicate to, engage with and involve their own demographic.

*“The young people are really excited [...] through gaining this perspective, they can clearly identify a need for their own creative skills and value within the world of science.”*

[Reform Radio project lead]

The AudioLab fostered a co-production approach building on the positive social identities of the young adult participants. Rather than being conceived of as ‘have nots’ through relative educational, social or educational disadvantage, The AudioLab empowered the participants as ‘having’ creative talent and opinions that matter to, and are useful to, science. The young adults, throughout the pilots, developed an understanding of their value in science communication and scientific research. All of the group were unaware of patient involvement in research opportunities at the outset. Beyond the pilots, several (three from second cohort, one from first) of them went on to become public contributors to scientific research. This demonstrated an effective way of ‘closing the loop’ between public engagement and active involvement in research.

### Emerging and diverse young adults as science communicators

Through their engagement, the young adults produced stimulating and innovative digital content about scientific research, which was well-received by their peers, the project team and wider audiences. The radio show produced by the 2017 cohort reached 2324 people through Facebook Live interactions, people engaged on the day of broadcast, listen agains and downloads (as of March 2018). The project team, including researchers and science communicators, were impressed with the quality of the questions being asked by the content, the novelty of the creative approaches, the high production values, the intelligence shown in background research carried out by young people (eg. Questioning online content) and the initiative in sourcing comment and interviews as part of their packages.

Because of the ‘aha’ moment during the 2016 AudioLab – the realisation amongst the young adults and the project team of the potential role of emerging and diverse creatives as science communicators – in 2017, we placed more emphasis developing this aspect of the pilot programme, and its evaluation. Young adults valued the role of science communication:

*“I think it’s important to engage people with science, because I think it’s, it surrounds us everywhere”*

*“[There’s] lots of different things going on around science that is extremely inspiring and really appeals to a younger generation so I really do think about it a bit differently now, even more so like, I knew there were other ways, of communicating subjects to people, but I hadn’t seen it in quite the way that we’ve seen it today”*

[Young adult responses, 2017].

They developed an appreciation of the skills needed to engage people with science and research, and could identify with some of these:

*“You need the ability to understand at least the broad overview of the science and the piece that you’re trying to communicate out, not necessarily all the specifics but at least how it affects other parts of the world and the ability to transfer that out in a good way that’s approachable to everyone.”*

*“I’ve learned how people use creativity to mix that into science.”*

[Young adult responses, 2017].

One emerging spoken word artist became fascinated with the effect of music on the brain and gene editing, and started their own research into the topic, laying down some tracks. Through continued mentoring following the pilots, four members of the second AudioLab cohort and one member of the first cohort went on to apply for, and receive funding, to work one-to-one with researchers to co-create engagement around the researcher’s work (see below, MixLab). Several of the young adults (in both cohorts) commented on the fact that they would be more open to seeking work in a science communication (eg. Science centre) or research-related setting or to including science as part of their own creative practice.

Interestingly, some young adults drew comparisons between science and social change. In particular, in the second cohort, several of the young adults were intrigued by aspects of scientific research (including for example neuroplasticity, genetic variation) that suggested that change is possible at a biological level.

*“Once you do something in your brain, and open a pathway, and you do it more and more and more, that pathway becomes easier, and once you don’t do, like, something, the pathway goes kind of thinner, and that was interesting.”*

[Young adult, 2017 cohort].

This is also exemplified by the refrain of the DNA song in the radio show:

*“One faulty gene isn’t always what it seems*

*It doesn’t have to stop you from following your dreams*

*Things might not always be what you expect them to be*

*Like bad things don’t always happen in patterns of three*

*Believe in yourself and the truth will set you free.”*

The project team were confident that the young adults had been effectively engaged with science. Reflecting on the aims of the project after the first AudioLab pilot, we had not expected that this project might produce creative communicators who would be less ‘scared’ of science as a subject area, and who might be more likely go on to produce future content around science. This represents an exciting potential for the project and could lead to more co-production of public engagement, and stimulate a diversity of voices in science communication. We reimagined our public not as an audience but as creative producers of public engagement.

### Interactions with researchers and science communicators

In 2016, six research scientists, one clinician and one patient were involved in the sessions (in addition to the project leads). In 2017, the group interacted with six external cultural, science & research organisations including Manchester Science Festival events, the Museum of Science and Industry, University of Salford. Ten research, science communication and engagement specialists (in addition to the two project leads) were involved in the sessions. Researchers and science communicators were keen, at the outset, to engage with audiences who might not see science as ‘for them’. They valued their interactions and working with the creative group.

Researchers became interested in the project through recognition of the need to engage more diverse audiences, but without the opportunity to do so - some *“didn’t know where to start”*. The AudioLab was therefore a welcome opportunity. Researchers rated the engagement of the young people with their area of science as high, valuing both the questions that the young people asked, and their creative talents. They were enthusiastic about the project; more senior researchers needed significant lead time in order to fit the project into their busy schedules. Some scientists had continued involvement throughout the pilots.

Interestingly, in the first cohort, the ‘taster’ for sleep research was presented on the science day by someone in a technical laboratory research role (other presenters were typically principal investigators or research group leads). The ‘status’ of the presenter, their young age, personal accessibility and relative public engagement inexperience - that they didn’t embody a traditional ‘expert’ - was an influence on the group’s choice of topic, in the opinion of the project leads. As a result, in 2017, the project leads carefully ensured that the researchers involved in The AudioLab represented different levels of seniority, as well as diversity in their ages, backgrounds, ethnicities, subject areas and skills. Our evaluation shows that interacting with scientists was a particular highlight of the pilot in 2017, amongst the young adults.

*“Getting to meet the scientists from different areas was very very pleasurable to me”.*

[Young adult, 2017 cohort].

One young adult further commented on the need for researchers to be inclusive in their engagement endeavours:

*“I believe you’ve got to be really really open minded and be able to touch base with lots of different people, and younger people, I believe that you shouldn’t have any kind of … what’s the word… you shouldn’t be like pretentious in any way about your knowledge or what you feel like you know as a sci… I’m not generalising in any way, because I don’t think everybody is like that, as a scientists but I just think that you’ve got to be really open to be a good communicator.”*

[Young adult, 2017 cohort].

Unanimously, the science communicators and public involvement specialists involved in The AudioLab project valued working with and hearing the opinions of the young adults. In particular, the Director of the Manchester Science Festival committed to work with some of the group in programming 2018’s Festival, in order to find ways to increase inclusion within the Festival.

### What happened next?

**Fig. 2:**
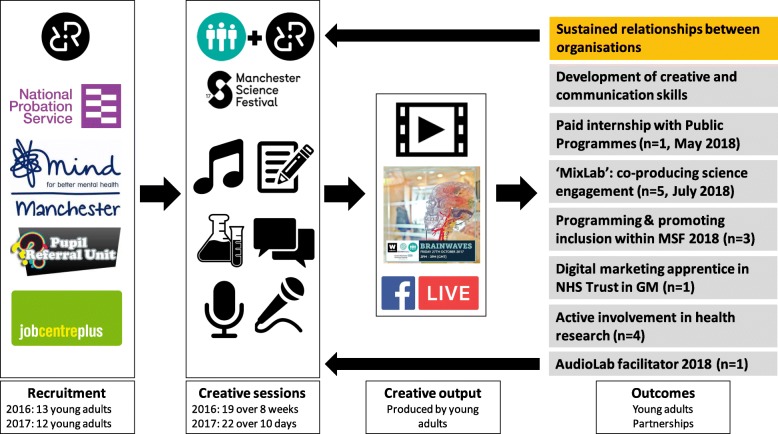
Summary of The AudioLab process, outputs and outcomes

Figure 2 summarises The AudioLab process, outputs and outcomes. Through continued contact and evaluation, progression routes for The AudioLab participants included:

- Four young adults (three from the 2017 cohort and one from the 2016 cohort) went on to seek and secure opportunities for active involvement in research (eg. Membership of a Young Person’s Advisory Group, membership of condition- or research-specific research user groups)
- Several further developed their creative and communication skills independently (eg. Through broadcast on other community radio stations, freelance videography, other public engagement projects)
- One young adult from the 2017 cohort undertook a 3-month paid internship with the Public Programmes team at Manchester University NHS Trust: a not-for-profit organisation specialising in public and patient involvement research (co-led by one of the co-authors of this paper)
- One young adult became a creative and digital marketing apprentice within an NHS Trust in Greater Manchester.
- Five (one from the 2016 cohort and four from the 2017 cohort) have been awarded funding (through the University of Manchester’s Wellcome Trust ISSF scheme) to work intensively 1:1 with scientists in a creative exchange, to co-produce engagement with a research topic through dance music and writing: the MixLab project happening in ‘co-production week’ in July 2018.
- Two young adults will work with Manchester Science Festival 2018 to help programme more inclusive activities as part of the Festival including through the assessment of projects wishing to be part of the Festival; another has been offered a slot on ‘Experimental Words’, a Festival event, pairing poets and scientists to co-create spoken word and/or performance around a scientific subject.
- One young adult will help facilitate the AudioLab in 2018, with a long-term view to leading the initiative.
- The 2017 cohort support each other through a What’s App group.

The progression routes undertaken by participants of The AudioLab are testament to their continued, supported, interest in science communication and public involvement in research, and demonstrate a route by which the audiences and producers of public involvement in scientific research can be diversified.

Progression routes for the project leads include:

- The Reform Radio project lead has had increased engagement with research organisations, for example through being part of a Community-University Partnership Initiative.
- The Public Programmes lead has developed their understanding and skills in working with young people from diverse backgrounds, and in working in partnership with social change and community organisations.

These points are expanded on below.

### Opportunities and limits of our evaluation approach

This was a complex project to evaluate, and the co-authors recognise the limitations of their evaluation approach. Working with young adults seeking employment is not conducive to traditional surveys as often such young adults can already feel very closely examined in their attempts to find employment or training, leading to feelings of ‘not another questionnaire’; the results of such surveys may therefore also be misleading. As previously discussed, establishing trust, openness and equal spaces for expression was a key element of the programme, especially at the start. Therefore we attempted to embed our evaluation as much as possible within the pilots themselves, using audio. In this way, evaluation questions could also provide opportunities to use the technical equipment, develop confidence in speaking and expression, and provide ‘talking points’ for the group. The inclusion, in 2017, of a summary evaluation from the point of view of one of the participants was an opportunistic approach, but one which the co-authors, and the participant, found valuable. It also demonstrates a creative approach to evaluation.

The AudioLab is a long-term partnership, including multiple relationships (between the young people themselves, between the participants and the project leads, and between the project leads themselves) which offer rich opportunities for evaluation (eg. The continued contact between the young people and the project leads beyond the pilot phases) and also merit deeper evaluation. We recognise the limits of self-evaluation and plan to include an external evaluator in some way in the future of The AudioLab.

### Personnel, ways of working, and partnership

Working with young adults seeking employment sometimes meant that sessions and group meetings were missed (eg. Because of ill health, or job interviews) which the project leads accepted as inevitable and designed the programme around. For example, the structure of the session days made sure that a piece of content was started and completed within a day, giving a sense of achievement, allowing sessions to be missed without missing out, as well as producing a huge variety of content. Establishing close contact and trusted relationships with the young adults, through dependable and daily presence and having “conversations that matter”, as well as maintaining the momentum of activity (especially in the second cohort) helped to sustain engagement.

*“Embracing the chaos”* became a theme of the project management, amongst the project leads. By working in partnership, co-producing with the young people and supporting their autonomy and direction, The AudioLab was not a linear process (despite how it might appear on this page), requiring ultimate flexibility and alternative thinking about how to ‘do’ public involvement in scientific research. At the outset, and during the partnership, strong shared values were key to the co-production process, including integrity, faith in the process, respect for each other and the young adults, a commitment to reflection and flexibility. The expertise, experience and relative confidence of the co-authors was also valuable in this regard. The project made use of the co-author’s significant experience of science communication and public engagement, as well as their existing connections with scientists and science communicators, through their networks and role as part of NHS and University research environments; it would have been hard to source researchers and science communicators for the project without these connections. Likewise, the co-author’s significant experience of working with young adults, applied theatre and applied radio methods, creative and participatory techniques was invaluable.

The project leads, front the outset, were committed to the need to maintain a relationship with the young adults beyond a ‘one-off’ experience. This supported several positive progression routes for the young adults. The relationships were upheld even when things didn’t turn out quite so right. For example, we continue to work with a participant who has been imprisoned.

It was important to invest in the relationship between the co-authors and their organisations too. Activities such as spending time together to understand each other’s work without time or target dependencies, joint conference attendance and dissemination activities embedded the partnership and its longevity. The Public Programmes co-author developed their understanding and appreciation of how a grass roots social change organisation works; the Reform Radio co-author developed their understanding and appreciation of how science works, and how research organisations work.

Such partnership investment and working takes resource. The co-author’s Fellowship was the enabler for this, financially and time-wise, allowing the co-author to take ‘time out’ of the day job, and provide funding for conference attendance etc. However, aspects of the partnership are now built in, commensurately, to the co-author’s employment and funding objectives to ensure the continuity of the relationship and programmes (the AudioLab will run year on year until at least 2022, through funding from the National Institute of Health Research Manchester Biomedical Research Centre and Clinical Research Facility and the Wellcome Trust). Being part of a vibrant City enabled the project leads to take advantage of cultural and research events and activities, especially the Manchester Science Festival; however, much was achieved through the ingenuity of the programme design and would not necessarily rely on such resource.

### Accessing research institutions

Reflecting on their partnership, the co-authors have repeatedly asked each other: How can we open more doors for social change organisations to work with research organisations? How do grass roots organisations know where the doors are? How do we shift power balances so we don’t have to rely on being invited over the threshold? How do we create more mediators (like the co-author) within research organisations to create and open doors (to step aside when more doors are open, or become invisible)? And while The AudioLab doesn’t answer all these questions, it provides evidence and a model for an alternative way of working to promote inclusion and social change. Other exciting opportunities are emerging in this area too (eg. National Coordinating Centre for Public Engagement Community-University partnerships scheme, INVOLVE co-production guidance).

### A transferable and scaleable model?

We believe that The AudioLab’s methodology is unique in working in partnership across social change and research organisations, to embed community engagement with science within existing structures and opportunities for young people seeking employment (and therefore also low-cost). We believe that this approach can be broadened to all scientific areas, and adapted for other settings. We believe it is sustainable by placing a co-production ethos and partnership approach (rather than a project-based approach) at its heart. The AudioLab is now embedded within a research and cultural offer in Greater Manchester, including, now, as an official partner of Manchester Science Festival.

Whilst it requires a degree of existing experience and expertise, we believe the model could be:

- Adopted by other public engagement and involvement groups or units within research organisations, working with social change organisations. We now hope to generate revenue for The AudioLab’s additional (small) running costs, by training other organisations to run similar programmes.
- Incorporated as an exemplar of alternative practice within public involvement training, learning and development (for example, the co-authors already present the work on the Manchester Metropolitan University and University of Salford Science Communication courses).

Both the above will also extend the reach and add to the impact of The AudioLab in the future; more importantly though, it will empower more diverse young adults as audiences and producers of public involvement in scientific research.

## Conclusion

The AudioLab project presents an innovative, effective and potentially transferable model for engaging and involving young adults in scientific research.
